# The clinical characteristics and management of paediatric pelvic fractures: a changing landscape based on skeletal maturity

**DOI:** 10.1007/s00068-022-02108-5

**Published:** 2022-10-03

**Authors:** Victor Lu, Shrav Gowrishankar, Zaki Arshad, Azeem Thahir, Jonathan Lenihan, Scott Mcdonald, Jaikirty Rawal, Peter Hull, Daud Chou, Andrew Carrothers

**Affiliations:** 1 School of Clinical Medicine, University of Cambridge, Cambridge, CB2 0SP UK; 2 Department of Trauma and Orthopaedics, Addenbrooke’s Hospital, Cambridge, CB2 0QQ UK; 3 Department of Radiology, Addenbrooke’s Hospital, Cambridge, CB2 0QQ UK; 4 Christ’s College, St. Andrew’s Street, Cambridge, CB2 3BU UK

**Keywords:** Paediatric fracture, Pelvic fracture, Trauma, Skeletal maturity, Torode

## Abstract

**Introduction:**

Paediatric pelvic fractures (PPFs) are uncommon but signify serious trauma. A comprehensive multidisciplinary approach is needed due to a high number of associated injuries. This study aims to retrospectively analyse PPFs over a 5-year period and evaluate how advancing skeletal maturity changes fracture patterns and management plans.

**Methods:**

The trauma database was retrospectively reviewed for pelvic fractures in patients aged ≤ 18 years. Radiographs and CT scans were used to classify pelvic injuries according to the modified Torode classification and determine the status of the triradiate cartilage (open: skeletally immature; closed: skeletally mature). Data collected also included the mechanism of injury, clinical and functional outcomes, and associated injuries. Logistic regression analysis was performed to identify risk factors for associated abdominal injuries.

**Results:**

65 PPFs (2.8% of paediatric trauma admissions during the study period) were classified as type I (3.1%), type II (7.7%), type IIIa (32.3%), type IIIb (38.5%), type IV (18.5%) according to the modified Torode classification. The mean age was 13.41 ± 3.82. Skeletally immature children were more likely to be hit by a motor vehicle as a pedestrian (*p* < 0.001), be intubated (*p* = 0.009), acquire Torode type II (*p* = 0.047) and rami fractures (*p* = 0.037), and receive chest (*p* = 0.005) and head injuries (*p* = 0.046). Skeletally immature children were also less likely to acquire Torode type IV fractures (*p* = 0.018), receive surgical treatment for their pelvic injuries (*p* = 0.036), and had a faster time to full weight bearing (*p* = 0.013). Pelvis AIS score ≥ 4 (OR 5.3; 95% CI 1.3–22.6; *p* = 0.023) and a pedestrian accident (OR 4.9; 95% CI 1.2–20.7; *p* = 0.030) were risk factors for associated abdominal injuries. There was a strong association between a higher pelvic fracture grade and the proportion of patients with closed triradiate cartilage (*p* = 0.036), hospital length of stay (*p* = 0.034), mean pelvic AIS score (*p* = 0.039), a pelvis AIS score of ≥ 4 (*p* = 0.022), mean ISS (*p* = 0.003), an ISS score between 25 and 75 (*p* = 0.004), average time to FWB (*p* = 0.001), requirement of blood products (*p* = 0.015), and a motor vehicle accident (*p* = 0.037).

**Conclusion:**

PPFs occurring in skeletally mature and immature patients are significantly different in terms of mechanism of injury, fracture severity, fracture pattern, and management strategy. There is a high rate of associated injuries, necessitating an integrated multidisciplinary approach in paediatric trauma centres.

## Introduction

Paediatric pelvic fractures (PPFs) are relatively uncommon, with a reported incidence ranging from 2 to 7.5% [1–4]. While the mechanism of injury varies, PPFs most commonly result from high-energy trauma, primarily due to motor vehicle accidents (MVAs) [5, 6]. Even in the category of MVAs, adult and paediatric pelvic fracture patterns tend to vary because of different mechanisms of injury (MOI).

Due to the forceful nature of these accidents, additional injuries often accompany pelvic fractures, with an average of five concomitant injuries being present [7]. In particular, in paediatric cases, injuries to the extremities [8] and genitourinary system [9] are common. The latter is attributed to the relative elasticity of the paediatric pelvis, which results in insufficient protection of the underlying viscera [10]. In contrast to adult cases, vascular haemorrhage in paediatric pelvic fractures is rare [8, 11]. Possible reasons include more effective vasoconstriction in the smaller vessels present in children, and the relative elasticity of the pelvic joints in paediatric patients, which makes single bone fractures more common—such fractures are associated with a decreased risk of haemorrhage from pelvic vessels [12, 13].

Reported mortality rates from pelvic injuries vary widely in this patient group, with reported rates being as low as 0.55% [7] to as high as 20% [14]. Chances of survival depend on the number and extent of other injuries sustained, with the presence of head and intrapelvic injuries increasing mortality risk [4, 15]. With regards to blunt trauma in children, central nervous system (CNS) injury is an important factor, and is associated with a tenfold increase in mortality risk compared to those without CNS injury [4]. However, there is a lack of data looking at mortality risk with particular reference to paediatric pelvic fractures, as opposed to general blunt trauma.

Overall, there is discordance in the literature on several findings reported on pelvic fractures in this age group. These include different findings on factors such as mortality rate, prevalence of fracture type, and the types of additional injuries associated with worse prognoses. The aim of this study is to perform a 5-years retrospective analysis of patients with paediatric pelvic fractures, looking at the factors discussed above, who were treated at a level one trauma centre. Factors assessed included general characteristics of the fracture, such as pelvic fracture type as confirmed by imaging, as well as additional injuries and mortality rate.

## Methods

After receiving approval from our institutional review board, the Trauma Audit and Research Network database, radiology database, and the local department database were retrospectively reviewed between January 2015 and January 2020 for all children admitted with a pelvic fracture.

To be included, patients were under 18 years of age, acquired a pelvic fracture from a blunt-force mechanism, and had adequate pelvic imaging for review. Those with firearm injuries were excluded. Data obtained from the retrospective review of complete medical records and radiographs were divided into four categories:

1. Epidemiological: Age, gender, MOI, survival, need for surgical orthopaedic procedures, hospital length of stay (LOS), intensive care unit (ICU) LOS.
2. Radiographical: Modified Torode classification, location of pelvic injury, status of triradiate cartilage.
3. Clinical and functional details: Injury severity score (ISS), pelvic abbreviated injury score (AIS), Glasgow coma scale (GCS) at the emergency department (ED), probability of survival, time to full weight bearing (FWB), need for intubation and blood products.
4. Associated non-trivial injuries divided into orthopaedic and non-orthopaedic injuries.

MOI was classified as a motor vehicle (MV) versus MV, MV versus pedestrian, slipped, fall from height, and others. Associated orthopaedic injuries included all non-pelvic bony fractures excluding facial and skull fractures. Associated non-orthopaedic injuries included abdominal trauma (*p*erforations, lacerations, ruptures of liver or spleen or bowel, abdominal aorta intima tear), chest trauma (*p*neumothorax, contusions, hemopneumothorax, lacerations), head injury (skull fracture, haematoma, intraventricular haemorrhage, pneumocephalus, cerebellum injury), and spinal injury (lamina fracture, facet dislocation, interspinous ligament laceration, transverse process fracture). The probability of survival (*p*s) was calculated using an online tool provided by the Trauma Audit and Research Network (TARN) [16]. A pelvic AIS ≥ 4 score was given for fractures with significant displacement with accompanying vascular damage and retroperitoneal haemorrhage.

All radiographs, including computerised tomography (CT) scans if available, were reviewed by two authors to classify pelvic fractures according to that proposed by Torode and Zeig [17]. CT scans were performed at the discretion of the consultant paediatric orthopaedic surgeon, for patients with clinical features of an unstable pelvis and persistent abdominal pain. The Torode classification divides pelvic fractures into four groups: (I) avulsion fractures, (II) iliac wing fractures, (III) simple ring fracture (stable), (IV) ring disruption (unstable) and pelvic fractures combined with acetabular fractures. Fracture stability was assessed by clinical and radiographical methodologies. Stable fractures had ≤ 2 mm fracture displacement on radiographs, were stable to pelvic compression, had no hip dislocations and no combined pelvic and acetabular fractures. The Torode classification was modified by Shore et al. who subdivided type III fractures into ‘A’ and ‘B’ [18]. The former (IIIa) is a stable anterior ring fracture, and the latter (IIIb) is a stable anterior and posterior ring fracture. The modified Torode PPF classification is predictive of morbidity and death in the multi-trauma setting [18], and was used in this study. Patients were divided into two groups based on skeletal maturity, which was assessed based on the status of the triradiate cartilage (open: immature; closed: mature).

Statistical analysis was performed using IBM SPSS Statistics version 27. Differences between the skeletally mature and immature cohorts were analysed. Categorical data were analysed by the chi-squared test or Fischer’s exact test if an expected value was below five. The Mann–Whitney *U* test was used for non-parametric continuous variables. Univariable analysis was performed to analyse the risk factors for outcomes of interest, from which the odds ratio of the outcome occurring for a given risk factor, 95% confidence interval (CI), and *p* value were obtained. Statistical significance was defined as *p* ≤ 0.05, and shown in bold in the results tables.

## Results

### Epidemiology

During the 5-year study period, there were 2340 paediatric trauma cases. There were 65 paediatric pelvic cases that met the inclusion criteria (mean age 13.41 ± 3.82), with 40 in the skeletally mature group (mean age 15.7 ± 1.25) and 25 in the skeletally immature group (mean age 9.74 ± 3.69), giving an overall incidence rate of 2.8%. Fifteen patients were brought to our institution via the East Anglian Air Ambulance. Table 1 compares the demographics, clinical presentation, and functional outcomes between skeletally mature and immature cohorts. Overall, the most common MOI was a pedestrian struck by a MV (41.5%). This was also the case for the skeletally immature cohort and occurred more frequently than in the skeletally mature cohort (68% vs 25%; *p* < 0.001). MV colliding with another MV occurred more often in the skeletally mature cohort (47.5% vs 16%; *p* = 0.010). Only one death was recorded within 30 days after the accident, in a skeletally immature nine-year-old male who suffered a subarachnoid haemorrhage and 16 mm deep-seated contusion inferior to the right basal ganglia after a collision with a car. The cause of death was a traumatic brain injury.

**Table 1:** Comparison between skeletally immature and mature cohorts

Patient characteristics	All patients (*n* = 65)	Immature (*n* = 25)	Mature (*n* = 40)	*p* value
Gender (male; female)	40; 25	18; 7	22; 18	0.171
Deceased, *n* (%)	1 (1.5)	1 (4)	0	0.202
Hospital LOS (days) ± SD	13.9 ± 18.9	12.04 ± 12.6	15.0 ± 22.0	0.695
ICU stay, n (%)	28 (43.1)	12 (48)	16 (40)	0.526
ICU LOS (days) ± SD	2.9 ± 5.3	3.8 ± 6.5	2.4 ± 4.4	0.403
Mean pelvic AIS score	3 ± 1.05	2.76 ± 0.97	3.15 ± 1.08	0.148
Pelvic AIS ≥ 4 (%)	26 (40)	7 (28)	19 (47.5)	0.118
ISS, *n* (%)				
Mean	28.92 ± 14.27	30.08 ± 15.87	28.2 ± 13.3	0.621
4–8	5 (7.7)	3 (12)	2 (5)	0.303
9–14	6 (9.2)	2 (8)	4 (10)	0.786
15–24	20 (30.8)	7 (28)	13 (32.5)	0.702
25–75	34 (52.3)	13 (52)	21 (52.5)	0.969
GCS (mean) ± SD	12.3 ± 4.2	11.16 ± 4.54	12.98 ± 3.81	0.119
Intubation (*n*, % yes)	19 (29.2)	12 (48)	7 (17.5)	**0.009**
Probability of survival (%) ± SD	91.7 ± 15.1	87.7 ± 17.5	94.1 ± 13.1	0.483
Average time to FWB (weeks) ± SD	4.7 ± 4.0	3.12 ± 2.88	5.7 ± 4.3	**0.013**
Blood products, *n* (%)	18 (27.7)	6 (24)	12 (30)	0.599
Non-pelvic Orthopaedic Operations, *n* (%)	23 (35.4)	8 (32)	15 (37.5)	0.652
Pelvic Operations, *n* (%)	12 (18.5)	2 (8)	12 (30)	**0.036**
Mechanism of injury				
MV vs MV	23 (35.4)	4 (16)	19 (47.5)	**0.010**
MV vs Pedestrian	27 (41.5)	17 (68)	10 (25)	**< 0.001**
Fall from height	6 (0.23)	2 (8)	4 (10)	0.786
Slipped	4 (6.15)	2 (8)	2 (5)	0.624
Other	5 (7.7)	0 (0)	5 (12.5)	0.164
Torode classification, *n* (%)				
I	2 (3.1)	0	2 (5)	0.256
II	5 (7.7)	4 (16)	1 (2.5)	**0.047**
IIIa	21 (32.3)	10 (40)	11 (27.5)	0.294
IIIb	25 (38.5)	10 (40)	15 (37.5)	0.840
IV	12 (18.5)	1 (4)	11 (27.5)	**0.018**
Associated injuries by region, *n* (%)				
Abdomen	11 (16.9)	5 (20)	6 (15)	0.601
Spine	9 (13.8)	4 (16)	5 (12.5)	0.691
Chest	25 (38.5)	15 (60)	10 (25)	**0.005**
Head	24 (36.9)	13 (52)	11 (27.5)	**0.046**
Non-pelvic skeletal fractures	41 (63.1)	15 (60)	26 (65)	0.684
Pelvic injury, *n* (%)				
Acetabular fracture	18 (27.7)	3 (12)	15 (37.5)	**0.025**
Rami fracture	31 (47.7)	16 (64)	15 (37.5)	**0.037**
Sacral fracture	11 (16.9)	6 (24)	5 (12.5)	0.229
Multiple pelvic fractures	22 (33.8)	10 (40)	12 (30)	0.407
Sacroiliac and pubis symphysis diastasis	15 (23.1)	7 (28)	8 (20)	0.456

*AIS* abbreviated injury scale, *MV* motor vehicle, *LOS* length of stay, *ICU* intensive care unit, *FWB* full weight bearing, *ISS* injury severity score, *SD* standard deviation; *﻿p* ≤ 0.05 values given in bold

Skeletally immature patients were less likely to receive operative treatment for non-pelvic orthopaedic fractures (32% vs 37.5%; *p* = 0.652), and pelvic fractures (8% vs 30%; *p* = 0.036). The two skeletally immature patients who needed surgery for pelvic fractures both had acetabular and posterior pelvic ring injuries. Two skeletally immature patients and six skeletally mature patients received operations for both pelvic and non-pelvic orthopaedic injuries. Both the hospital LOS, percentage needing admission to ICU, and ICU LOS were longer in the skeletally immature cohort (15.64 days vs 15.0 days; 68% vs 47.5%; 3.64 vs 3.28 days, respectively), but none were statistically significant.

### Radiology

**Fig. 1:**
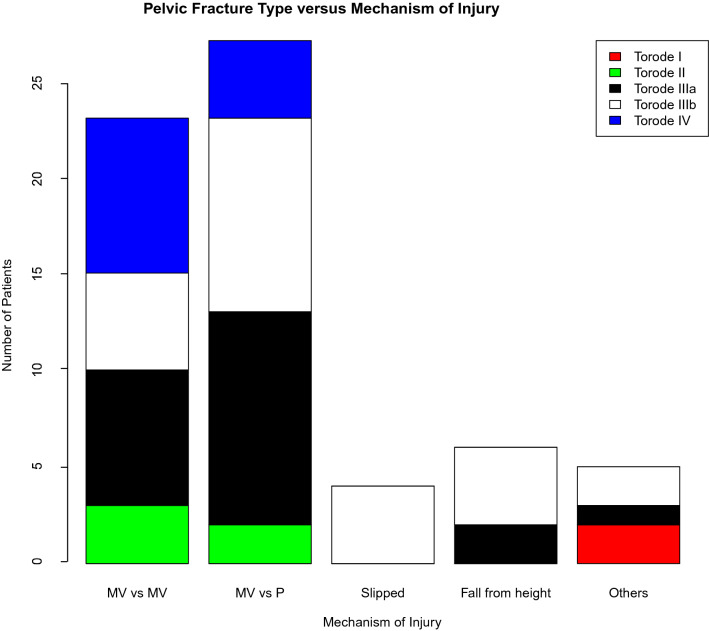
Pelvic fracture type versus mechanism of injury

The distribution of fractures according to the modified Torode classification presented against MOI is shown in Fig. 1. Table 2 compares the demographics, clinical presentation, and functional outcome between different grades of pelvic fractures. Skeletally immature patients were more likely to obtain type II fractures (16% vs 2.5%; *p* = 0.047) and less likely to obtain type IV fractures (4% vs 27.5%; *p* = 0.018). There was a strong association between a higher pelvic fracture grade and the proportion of patients with closed triradiate cartilage (*p* = 0.036), hospital LOS (*p* = 0.034), mean pelvic AIS score (*p* = 0.039), a pelvis AIS score of ≥ 4 (*p* = 0.022), mean ISS (*p* = 0.003), an ISS score between 25 and 75 (*p* = 0.004), average time to FWB (*p* = 0.001), requirement of blood products (*p* = 0.015), and encountering an MV versus MV accident (*p* = 0.037) (Table 2). Those with higher pelvic fracture grade also had a lower probability of survival (*p* = 0.601), higher chance of ICU admissions (*p* = 0.124), longer ICU stay (*p* = 0.114), and older age (*p* = 0.051) but they did not reach statistical significance.

**Table 2:** Comparison based on modified Torode classification

Patient Characteristics	Torode I (*n* = 2)	Torode II (*n* = 5)	Torode IIIa (*n* = 21)	Torode IIIb (*n* = 25)	Torode IV (*n* = 12)	*p* value
Age	14.5 ± 0.7	10.4 ± 4.4	13.4 ± 3.3	12.8 ± 4.5	15.7 ± 2.0	0.051
Gender (male; female)	2; 0	4; 1	12; 9	16; 9	6; 6	0.578
Deceased, n (%)	0 (0)	0 (0)	1 (4.8)	0 (0)	0 (0)	0.718
Skeletal maturity (n, % closed triradiate cartilage)	0 (0)	1 (20)	11 (52.4)	15 (60)	11 (91.7)	**0.036**
Hospital LOS (days) ± SD	0 (0)	10.2 ± 5.7	13.5 ± 18.3	13.2 ± 20.0	19.8 ± 22.2	**0.034**
ICU stay, n (%)	0 (0)	1 (20)	6 (28.6)	14 (56)	7 (58.3)	0.124
ICU LOS (days) ± SD	0 (0)	5 ± 11.2	1.3 ± 2.9	3.1 ± 4.5	5.1 ± 6.7	0.114
Mean pelvic AIS score	2 ± 0	3 ± 1	2.6 ± 0.9	3.0 ± 1.0	3.8 ± 1.1	**0.039**
Pelvic AIS ≥ 4 (%)	0 (0)	2 (40)	4 (19)	11 (44)	9 (75)	**0.022**
ISS, *n* (%)						
Mean	12 ± 5.7	19.6 ± 5.1	23.5 ± 14.1	32.0 ± 13.7	38.8 ± 11.3	**0.003**
4–8	1 (50)	0 (0)	4 (19)	0 (0)	0 (0)	0.065
9–14	1 (50)	1 (20)	3 (14.3)	1 (4)	0 (0)	0.119
15–24	0 (0)	3 (60)	7 (33.3)	9 (36)	1 (8.3)	0.191
25–75	0 (0)	1 (20)	7 (33.3)	15 (60)	11 (91.7)	**0.004**
GCS (mean) ± SD	15 ± 0	12 ± 4.2	11.3 ± 4.9	12.7 ± 3.7	12.7 ± 4.2	0.707
Intubation (*n*, % yes)	0 (0)	2 (40)	7 (33.3)	8 (32)	2 (16.7)	0.683
Probability of survival (%) ± SD	99.65 ± 0.18	95.0 ± 5.9	92.0 ± 17.2	92.4 ± 12.4	90.4 ± 20.5	0.601
Average time to FWB (weeks) ± SD	0 ± 0	1.6 ± 2.6	4.8 ± 4.3	3.9 ± 3.1	8.3 ± 3.4	**0.001**
Blood products, *n* (%)	0 (0)	2 (40)	1 (4.8)	8 (32)	7 (58.3)	**0.014**
Non-pelvic orthopaedic operations, *n* (%)	1 (50)	3 (60)	9 (42.9)	6 (24)	4 (33.3)	0.483
Pelvic operations, *n* (%)	1 (50)	1 (20)	4 (19.0)	3 (12)	5 (41.7)	0.261
Mechanism of injury						
MV vs MV	0 (0)	3 (60)	7 (33.3)	5 (20)	8 (67.7)	**0.037**
MV vs Pedestrian	0 (0)	2 (40)	11 (52.4)	10 (40)	4 (33.3)	0.592
Fall from height	0 (0)	0	2 (9.5)	4 (16)	0 (0)	0.509
Slipped	0 (0)	0	0 (0)	4 (16)	0 (0)	0.146
Other	2 (100)	0	1 (4.8)	4 (16)	0 (0)	0.078
Associated injuries by region, *n* (%)						
Abdomen	0 (0)	2 (40)	2 (9.5)	5 (20)	2 (16.7)	0.511
Spine	1 (50)	1 (20)	1 (4.8)	6 (24)	0 (0)	0.096
Chest	2 (50)	3 (60)	7 (33.3)	11 (44)	2 (16.7)	0.128
Head	0 (0)	3 (60)	11 (52.4)	8 (32)	2 (16.7)	0.144
Non-pelvic skeletal fractures	1 (50)	4 (80)	12 (57.1)	16 (64)	8 (66.7)	0.885
Pelvic injury, *n* (%)						
Acetabular fracture	0 (0)	1 (20)	7 (33.3)	6 (24)	4 (33.3)	0.807
Rami fracture	0 (0)	1 (20)	13 (61.9)	14 (56)	3 (25)	0.084
Sacral fracture	1 (50)	1 (20)	2 (9.5)	5 (20)	2 (16.7)	0.631
Multiple pelvic fractures	0 (0)	1 (20)	9 (42.9)	7 (28)	5 (41.7)	0.571
Sacroiliac and pubis symphysis diastasis	1 (50)	2 (40)	2 (9.5)	6 (24)	4 (33.3)	0.340

*﻿p* ≤ 0.05 values given in bold

Compared to Torode IIIa fractures, those with Torode IIIb fractures had over a six-fold greater frequency of requiring blood products than Torode IIIa fractures (32% vs 4.8%), nearly twice the frequency of being admitted to ICU (56% vs 28.6%), over twice the length of stay in ICU (3.1 vs 1.3 days), and over twice the frequency of having a ≥ 4 pelvic AIS score (44% vs 19%).

Regarding pelvic injury patterns, rami fractures were the most common overall (47.7%), both in the skeletally immature (64%) and mature (37.5%) subgroups. Compared to skeletally mature patients, skeletally immature patients were more likely to acquire rami fractures (64% vs 37.5%; *p* = 0.037) and less likely to acquire acetabular fractures (12% vs 37.5%; *p* = 0.025).

### Clinical and functional details

In both the skeletally mature and immature cohorts, the majority of patients had an ISS score between 25 and 75. Using the commonly defined criteria for major trauma with a threshold ISS score of 15 [19], 83.1% of patients fell within this category, with a similar percentage in both the skeletally immature (80%) and mature cohorts (85%). The mean pelvic AIS was 3.03 (SD 1.0), with 26 patients (40%) having a ≥ 4 AIS score. The average time to FWB was longer in the skeletally mature cohort compared to the immature cohort (5.7 vs 3.1 weeks; *p* = 0.013). A higher proportion of skeletally immature patients required intubation (48% vs 17.5%; *p* = 0.009).

### Associated injuries

**Fig. 2:**
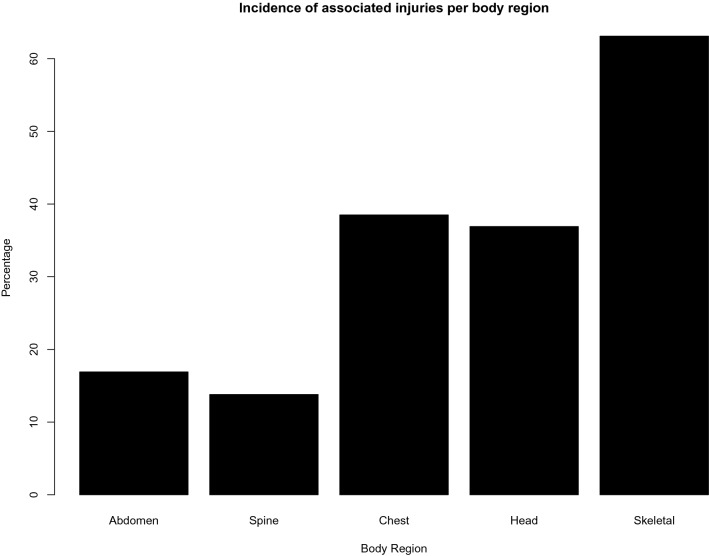
Incidence of associated injuries per body region

Associated injuries were a common occurrence (Fig. 2). Overall, non-pelvic skeletal injuries were the most common (63.1%). There were significantly more chest (60% vs 25%; *p* = 0.005) and head injuries (52% vs 27.5%; *p* = 0.046) in the skeletally immature cohort. A pelvis AIS score ≥ 4 (OR 5.3; 95% CI 1.3–22.6; *p* = 0.023) and a collision between pedestrian and MV (OR 4.9; 95% CI 1.2–20.7; *p* = 0.030) were risk factors for associated abdominal injuries (Table 3).

**Table 3:** Risk factors for associated abdominal injuries

*N* = *65*	Associated abdominal injuries (*n*, %)	Odds ratio (95% CI)	*p* value
Gender			
Male	6/40	0.706 (0.191–2.614)	0.602
Female	5/25		
MV versus MV			
Yes	3/23	0.638 (0.151–2.683)	0.539
No	8/42		
MV versus Pedestrian			
Yes	8.27	4.912 (1.164–20.726)	**0.030**
No	3/38		
Slipped			
Yes	1/4	1.700 (0.160–1.805)	0.660
No	10/61		
Fall from height			
Yes	1/6	0.980 (0.103–9.318)	0.986
No	10/59		
Pelvis AIS ≥ 4			
Yes	7/25	5.333 (1.260–22.567)	**0.023**
No	4/40		
Torode classification			
I	0/2	0.974 (0.498–1.903)	0.937
II	2/5		
IIIa	2/21		
IIIb	5/25		
IV	2/12		

*MV* motor vehicle, *AIS* abbreviated injury scale; *﻿p* ≤ 0.05 values given in bold

Amongst the non-pelvic skeletal injuries, lower limb fractures were the most common (40%). Skeletally immature patients (*p* = 0.023) and those with a higher pelvic fracture grade (*p* = 0.042) were more likely to acquire femur fractures (Tables 4, 5).

**Table 4:** Distribution of non-pelvic skeletal fractures according to skeletal maturity

Patient characteristics	All patients (*n* = 65)	Immature (*n* = 25)	Mature (*n* = 40)	*p* value
Upper limb fracture	16 (24.6)	6 (24)	10 (25)	0.927
Humerus	6 (9.23)	1 (4)	5 (10.25)	0.249
Radius/ulna	10 (15.4)	5 (20)	5 (12.5)	0.415
Lower limb fracture	26 (40)	13 (52)	13 (32.5)	0.118
Femur fracture	16 (24.6)	10 (40)	6 (15)	**0.023**
Tibia/fibula fracture	9 (13.8)	6 (24)	3 (7.5)	0.061
Foot	4 (6.15)	1 (4)	3 (7.5)	0.568
Clavicle	7 (10.8)	2 (8)	5 (12.5)	0.569
Rib	4 (6.2)	1 (4)	3 (7.5)	0.568

*﻿p* ≤ 0.05 values given in bold

**Table 5:** Distribution of non-pelvic skeletal fractures according to Torode classification

Patient Characteristics	Torode I (*n* = 2)	Torode II (*n* = 5)	Torode IIIa (*n* = 21)	Torode IIIb (*n* = 25)	Torode IV (*n* = 12)	*p* value
Upper limb fracture	1	1 (20)	4	7	3	0.868
Humerus	1	0 (0)	2	3	0	0.205
Radius/ulna	0	1 (20)	2	4	3	0.762
Lower limb fracture	0	4 (80)	10	8	4	0.194
Femur fracture	0	1 (20)	3 (14.3)	4 (16)	3 (25)	**0.042**
Tibia/fibula fracture	0	3 (60)	4	3	1	0.856
Foot	0	0 (0)	3	1	0	0.427
Clavicle	0	1 (20)	1	3	2	0.745
Rib	0	0 (0)	1	1	2	0.553

*﻿p* ≤ 0.05 values given in bold

## Discussion

Pelvic fractures are usually a consequence of high-energy trauma and are uncommon amongst the paediatric population. In this single-centre study over a 5-years period, PPFs corresponded to 2.8% of our paediatric trauma admissions. The literature only provides a vague indication of the incidence of PPFs [20]. Our incidence rate is lower than a recent study over a 14-years time period which suggested 3.5% [5] but greater than an earlier study from a large paediatric hospital which suggested a rate of 10 PPFs per year [21]. The actual rate is likely to be higher since one study reported that 93% of post-mortem assessments of paediatric patients that died from blunt trauma had pelvic polyfractures [22]. The main findings of this study include the predominance of motor vehicle accidents as an MOI, male gender, and non-pelvic skeletal fractures being the most commonly encountered associated injury. Compared to their skeletally mature counterparts, skeletally immature children were more likely to be hit by a motor vehicle as a pedestrian, be intubated, acquire Torode type II and rami fractures, and receive chest and head injuries. Skeletally immature children were also less likely to acquire Torode type IV fractures, receive surgical treatment for their pelvic injuries, and had a faster time to FWB. Furthermore, the mortality rate, GCS score, and average probability of survival did not significantly differ depending on skeletal maturity or severity of fracture.

Parameters that can assess skeletal maturity include the status of the triradiate cartilage, Risser classification, greater trochanteric apophysis closure, and proximal femoral apophysis closure. Since this is a study on paediatric pelvic fracture, a parameter based on the pelvis was deemed most appropriate. The Risser classification has limitations that complicates accurate staging, such as failing to take into account anomalous ossification of the iliac apophysis [23]. This can be overcome by bending anteroposterior (AP) pelvic films, but may not be suitable for the younger paediatric cohort [24]. Recent studies have suggested dividing Risser stage 0 into two groups based on triradiate cartilage status [25]. Hence, we deemed an assessment of the triradiate cartilage on radiographs to be the most reliable for determining skeletal maturity. The age at which the triradiate cartilage closes is clinically important because the mechanical properties of the pelvis changes, namely a loss in elasticity with skeletal maturity [18]. There is a general consensus amongst studies that this is 14 for males and 12 for females [26]. The transition point could distinguish paediatric from adult patients, given the different treatment choices and outcomes between the two cohorts. Skeletally mature patients were more likely to sustain Torode type IV fractures (*p* = 0.018) and less likely to acquire type II fractures (*p* = 0.047). Importantly, skeletally mature patients were more likely to receive operative treatment for both pelvic (*p* = 0.036) and non-pelvic injuries (*p* = 0.652). Karunakar et al. suggested that skeletally mature patients with unstable pelvic and acetabular fractures should be managed in the same manner as adults, namely by operative fixation [27]. Even in immature patients, Karunakar et al. showed successful operative management, but the clinical indications are less clear, perhaps warranted for pelvic fractures that distort the skeletally immature pelvis [27]. The conservative treatment of fifteen skeletally immature patients with unstable pelvic fractures by McDonald et al. led to limb length discrepancy, triradiate and sacroiliac growth arrests [28]. Indeed, patients in our cohort with type IV fractures were more likely to receive operative treatment (*p* = 0.261). However, reports of pelvic growth arrest and bony remodelling after the operative treatment of skeletally immature patients [5], and the loss of pelvic elasticity in females affecting future pregnancies may warrant future research into the optimal treatment modality.

MVAs accounted for the majority of injuries in our cohort (76.9%), agreeing with earlier studies [6, 29, 30]. Studies on adult pelvic fractures have also reported MVAs to be the most common MOI [26, 31]. Despite the high proportion of MVAs, none resulted in mortality in our cohort. Early studies have suggested a mortality rate as high as 25% [32], a figure higher than the 17.5% mortality rate reported for adult patients [33, 34], and suggested a significant number suffered from pelvic haemorrhage which mirrors the situation in adults. Yet our cohort and other recent studies disprove that. Adult pelvic fractures usually involve an ‘open book’ injury type created by an AP-directed force, leading to increased pelvic volume and fatal pelvic haemorrhage [26]. On the contrary, especially with the younger skeletally immature cohort who are more likely to be pedestrian struck (*p* < 0.001), a lateral compression force is experienced which is less likely to lead to pelvic haemorrhage [35]. Nearly half our cohort (47.7%) suffered from rami fractures, which was seen more frequently in the skeletally immature group (*p* = 0.037), indicating a lateral compression type of injury. Saglam et al. reported a ten-fold greater incidence of pedestrian crashes than MV crashes alone [36]. Furthermore, paediatric blood vessels have decreased vascular stiffness, increased endothelial function, and greater vasoactive properties, allowing a more robust vasoconstrictive response compared to their adult counterparts [37].

Despite the mortality rate in our cohort (1.5%) being lower than that in the recent literature [5, 6, 11, 30, 31, 35, 37], our mean ISS score (28.9) and proportion of Torode type IV fractures (18.5%) was not significantly different from recent studies [5, 11, 18, 35]. Nevertheless, Shaath et al. had 44.9% type IV fractures but only a 6% death rate [30]. The rate of paediatric unstable pelvic fractures (type IV) is lower than the adult population [3, 38], which could be due to the increased likelihood of the adult pelvis to break in multiple locations like a ‘pretzel’ [29]. Compared to bone and surrounding ligaments, the intrinsic weakness of the epiphyseal and apophyseal cartilage in children increases the chance of an avulsion fracture through the growth plate, which could lead to limb length discrepancy [39]. They have a high capacity for energy absorption and when not displaced, the damage can be hard to pick up on radiographs, with some suggesting that a minimum follow-up time of one year is needed for diagnosis [40].

Whilst PPFs per se are not necessarily life-threatening, they are indicative of serious trauma. With the greater flexibility of sacroiliac joints and malleability of the paediatric skeleton [35], greater forces are absorbed for a given pelvic fracture compared to adults. Hence, PPFs are often accompanied by concomitant injuries which can lead to mortality or long-term morbidity if not identified and treated promptly [41]. Mortality in PPF patients can be associated with many factors, such as decreased GCS, higher ISS scores, longer time spent in ICU, and a higher head AIS score. The only patient that died in our study suffered a traumatic brain injury. Nevertheless, no relationship between Torode grade and mortality was found (*p* = 0.718), agreeing with previous studies [5, 31, 33]. Other than additional skeletal injuries, chest and head injuries were the most prevalent, identified in 38.5% and 36.9%, respectively. This is in line with earlier studies that noted an increased involvement of the upper trunk, especially with more severe injuries [6, 31, 42, 43]. Chest injuries however occur less frequently than in the adult population, perhaps due to the greater compliance of a child’s chest wall [44].

Studies have pointed out that patients with intra-abdominal injuries, especially hollow viscus perforations, are sometimes missed, with difficulty to interpret signs in a traumatised child [3, 45]. This is concerning given that the clinical course in patients with concomitant pelvic/abdominal injuries is significantly longer [46], and the poorly developed abdominal wall musculature in children leads to a higher chance of injury than adults [44]. Our cohort had a 16.9% incidence rate of intra-abdominal injuries, which did not significantly differ between the skeletally mature and immature cohorts. Bond et al. reported a 20% rate, which could be due to their exclusion of Torode type I avulsion fractures [47]. Risk factor analysis for associated abdominal injuries revealed an important relationship with pelvis AIS score ≥ 4 (OR 5.3; 95% CI 1.3–22.6; *p* = 0.023), which agrees with the findings of Demetriades et al. [3]. Regarding non-pelvic skeletal injuries, lower limb fractures predominated (40%), with the incidence of femoral fractures equivalent to the incidence of upper limb fractures (24.6%). Intriguingly, the rate of femoral fractures is higher in the skeletally immature cohort and in those with a higher Torode grade. This could be due to the predominant MOI of skeletally immature patients, which is being pedestrian struck.

Our centre is a specialised adult and paediatric trauma centre, with the resources to prioritise treatment of critical trauma at risk of mortality. The main limitation of this study is the retrospective design and low patient numbers. The scarcity of these cases emphasises the need for multicentre studies or the establishment of national networks that deal with paediatric pelvic trauma management. The inclusion of patients 18 years or under was based on the World Health Organisation’s cut-off age for paediatric patients, despite evidence suggesting that there cannot be a single cut-off point for when a paediatric patient becomes an adult patient, but rather a gradual transition depending on multiple factors, such as skeletal maturity. The follow-up times varied widely, meaning that no conclusions can be drawn from this study about long-term results. This is important to investigate in future studies, especially in cohorts with a large group of skeletally immature patients, since the pelvis can malunite during growth [48]. Furthermore, studies could also incorporate a health economics analysis, to provide a more efficient specialist paediatric pelvic trauma service in tertiary centres.

## Conclusions

PPFs are a reliable hallmark of severe trauma in children and our study suggests the importance of early identification of associated trauma such as abdominal injuries which can be overlooked. A clinically useful classification system such as the modified Torode classification should be used to differentiate patients according to the severity of trauma, guide clinician’s understanding of the disease process, and aid treatment decisions. Patients with closed triradiate cartilage can be treated like adults, as they have similar MOIs, need for operative management, and fracture patterns. Those with open triradiate cartilage are unique, with significant differences in terms of MOI, management strategy, fracture severity and patterns. With improved intensive care and trauma management, mortality from pelvic fractures can be kept at a low level.
